# Brave New (Microbial) World: implications for nasal and sinus disorders^☆^

**DOI:** 10.1016/j.bjorl.2019.09.001

**Published:** 2019-09-12

**Authors:** Martin Desrosiers, Fabiana Cardoso Pereira Valera

**Affiliations:** a Centre Hospitalier de l’Université de Montréal (CHUM), Montreal, Canada; b Universidade de São Paulo (USP), Faculdade de Medicina de Ribeirão Preto, Ribeirão Preto, SP, Brazil; c Universidade de São Paulo (USP), São Paulo, SP, Brazil; d Université de Montréal, Montreal, Canada

## Emergence of microbiome - a new role for bacteria

Increased appreciation of the role of the microbiome in human health and disease is contributing to a revolution in our understanding of chronic diseases and has important implications for physicians.

While traditional physician training concentrates on management of acute infection and its attendant complications, the important role of bacteria and pathogens in chronic disease is increasingly appreciated, often in unexpected fashion. Over the past decade there has been an increased understanding of the importance of the bacterial communities present on all body surfaces and cavities.^1^ These bacterial communities, consisting of trillions of individual bacteria form different species, form bacterial communities of interacting bacterial species which we term the microbiome. Fuelled by the revolution in sequencing technology, researchers now use specific DNA signatures to identify bacterial organisms without having to culture them in the laboratory using conventional methods. These “culture independent” techniques allow identification of bacteria which grow poorly in culture conditions and have opened up a new treasure trove of knowledge on how bacteria contribute to development and persistent of chronic diseases.

It is now understood that every surface and cavity of the body contains its own specific microbiome, Early work began in the gut, with the realisation that altered bacteria balance in the microbiome (microbiome “dysbiosis”) contributes to persistence of disease. Surprisingly, this has been not so much about forming an abundance of pathogens and attendant toxins, but more a lack (loss) of healthy bacteria required for the maintenance of health.

Our understanding of how bacteria paradoxically contribute to health maintenance is growing exponentially. “Healthy” bacteria are associated with regulation of immune responses, defense against pathogenetic bacteria, and epithelial regeneration or repair of epithelial surfaces.^2^ Proof of the importance of these ‘healthy’ flora is offered by the finding that mice raised in a sterile environment show spontaneous inflammation of the gut. In addition to local effects, these bacteria may also secrete small molecules which travel via the blood stream to influence distant organs such as the brain. This has led to the appreciation that microbiome- mediated effects may be occurring locally, such as in the colon and inflammatory changes therein, but may also be inciting effects at as distance, such as in the brain. This has helped understand how changes in the microbiome in the gut may contribute to obesity, metabolic syndrome, Parkinson’s, and autism, to name only a few. Intriguingly, effects on medical therapy are also being suggested, with microbiome-induced modifications in drug metabolism altering responses to therapeutic agents in Parkinson’s disease and immune checkpoint inhibitor therapy for cancer also being suspected.^3^

In ENT, the role of the microbiome in maintenance of health is only beginning to be appreciated.^4^, ^5^ It has been understood for over a decade that bacteria play a role in the persistence of chronic disease, with Staphylococcus Aureus implicated via roles in biofilm formation, development of inflammation via superantigen production, and most recently, impairment of epithelial repair and regeneration. However, it is only recently revealed that chronic nasal and sinus ailments such as chronic sinusitis and nasal allergies are characterised by not only the presence of selected pathogens but also by a lack of healthy bacteria. In addition, it is also suggested that lack of acquisition of healthy bacteria in early life may predispose to development of allergies. Lack of exposure to maternal bacteria during Caesarian-section as opposed to vaginal delivery increases the risk of allergy in offspring.^6^ Further support for this is the finding that the lesser incidence of allergies in children raised in farming environments is accompanied by a strikingly different microbiome to allergic, urban-dwelling children.^7^ In these instances, this is suspected to be a more direct effect of bacteria directly in the nasal passages and sinuses, rather than distant effects from gut bacteria.

## Therapeutic manipulation of the microbiome

Taken together, these findings suggest the possibility of improving health via restoration or supplementation of healthy bacteria rather than focussing on pathogenic ones. However, for the moment, technologies for modulating or supplementing the microbiome remain a work in progress and the practitioner may have difficulty selecting between them.

The first example of microbiome supplementation therapy remains the “stool transplant”. “Yellow soup” made from feces collected from a healthy donor were suggested as treatment of diarrhea as far back as the time of Hippocrates.^8^ Over the past decade, this has been used clinically for episodes of *Clostridium difficile* colitis, with often life-saving effects. However, in addition to obvious psychological obstacles, lack of a standardised method or commercially available formulation pose the risk of transmitting infections from donor to recipient. This has led the FDA in the USA to restrict stool transplants only to patients with *C Difficile* diarrhea.^9^, ^10^

Given the difficulties with directly transferring bacteria from patient to patient, efforts have mostly concentrated on the use of *Probiotics*. Probiotics are defined by the World Health Organisation as ‘live micro-organisms’ that confer digestive and immune health benefits to the in taker when consumed in adequate amounts.” These bacteria have generally been used in food or cheese production for over a century, and usually benefit from ‘Generally Accepted as Safe’ status given their long history of safe use in industry.

Probiotics exert their beneficial effects by modulating inflammation, secreting small molecules which may act at a distance, and by restricting the growth of pathogenic bacteria via both direct bacterial inhibition and also via competition for scarce nutriments. Certain strains have beneficial effects upon epithelial regeneration and repair.^11^ Not all bacteria possess probiotic properties: changing even a few amino acid sequences in their DNA may alter them considerably. It is thus important to use isolates with documented probiotic properties and explains why manufacturers frequently identify their strain of bacteria specifically with letters or a code: all bacteria are not created equal.

Physicians may also hear the terms prebiotic or postbiotic. These refer either to substances such as dietary fiber, which when ingested encourage the growth of ‘healthy’ gut bacteria (pre-biotic) or else the health-conferring small molecules secreted by ‘healthy’ bacteria (post-biotic). This area remains in the early stages of development and it remains difficult to make firm recommendations for their use.

## Probiotics for the sinus?

The nasal passages and sinus cavities are good potential candidates for probiotic therapy.^12^, ^13^ Multiple authors have now documented microbiome dysbiosis in chronic rhinosinusitis and nasal allergy, leading several authors to suggest a role for microbiome manipulation in these disorders. While proponents of the ‘SNOT’ transplant suggest that direct transfer of material from healthy to diseased individuals may be possible,^14^ as in the stool transplant, practical limitations regarding risk of transmission of infection remain a limit to this approach. For the moment, attention has focussed on probiotics.

As orally administered probiotics for allergy and CRS have met with limited success at best,^15^ attention has mostly focussed on topical intranasal application of probiotics directly to the nasal passages via nasal spray or irrigation. In mice experiments, Lynch et al reported that application of Lactobacillus sakei prevented overgrowth of the purported pathogen *Corynebacterium Tuberculostereaticum*.^16^ However, these studies have not progressed to human trials.

The safety of this approach is supported by previous experiments. Mårtensson (2016)^17^ initially applied a nasal spray containing 40 Million (106) CFU (colony forming units, representing number of bacteria) lactic acid bacilli isolated from the honeybee to healthy volunteers and demonstrated the absence of ill effects with therapy. In a follow up study, they applied the same dosage to patients with CRS without nasal polyps.^18^ While there was no beneficial therapeutic effect noted, again no adverse effects were seen.

A beneficial effect of probiotics for nasal disease is nevertheless suggested by a clinical trial of probiotic Lactococcus lactis W136 in patients with chronic sinusitis with and without nasal polyposis unresponsive to treatment despite previous sinus surgery.^19^ In this trial, which was performed by our group, 24 patients received 1.2 billion CFU of L lactis W136 self-applied directly to the nasal and sinus passages via nasal and sinus irrigation. Treatment was twice-daily for fourteen days. Therapy was well tolerated and led to improvements in symptoms, measures of quality of life, and improvement in endoscopic scores. Gene expression profiling to identify implicated mechanisms suggested enhanced epithelial repair and regeneration, and modulation of inflammation. Microbiome profiling using 16 s technology showed reduction in the pathogens *Staphylococcus Aureus*, *Peptostreptococcus*, and *Enterobacteriacae*.

Explanation of the difference in results between the two trials may be explained by differences in bacteria used and experimental design. While L lactis W136 is a cocci, or spherical-shaped bacteria, the Mårtensson trial used a bacillus, or rod-shaped bacteria that differs from the normal flora of the nose, which are mainly cocci. In addition, the dosage used was 30x less than that used for the *L lactis W136* trial, and administered via a nasal spray, which has less intranasal penetration and deposition than does the nasal irrigation use in the *L lactis W136* trial. It is thus possible that these factors limited success of the Mårtensson trial.

## Microbiome therapy for the nose and sinuses: current and future perspectives

For the moment, direct applications of the implications of the nasal and sinus microbiome remain limited. Oral probiotics do not demonstrate great efficiency and the ‘snot transplant’ remains plagued by the same safety concerns as the stool transplant. The best option for the moment remains topical application of probiotic. Only one specifically formulated intranasal therapy is currently available, unfortunately only in North America. Based on probiotic *Lactococcus lactis W136*, Probiorinse™ Probiotic Nasal and Sinus Rinse is Health Canada approved as a natural product for relief of chronic sinusitis, nasal polyposis, nasal obstruction and nasal allergies, and has been on sale in the USA and Canada for fifteen months (www.probiorinse.com, www.probiorinse.ca). Post-marketing experience has shown favorable consumer acceptance and an absence of notable adverse effects.

The future of microbiome therapy is difficult to predict as numerous means of manipulating the microbiome are being suggested, including a potential use in prevention and treatment of the common cold. However, these will require confirmation of efficacy with clinical trials. A look into the crystal ball nevertheless suggests that continuous advances in sequencing technology will allow inexpensive analysis of individual patient microbiomes and genetic makeup, opening the future to ‘personalised’ therapeutic recommendations for enhanced results.

## Funding

### Martin Desrosiers:

Financial disclosures: President, Probionase Therapies Inc.

Funding: Merck, Sharpe & Dohme Corp./McGill Faculty of Medicine Grant for Translational: “Immune modulatory bacteria for the treatment of chronic rhinosinusitis”.

Fondation Antoine-Turmel, a private philanthropic organisation.

### Fabiana Cardoso Pereira Valera:

Research support: FAPESP, Sao Paulo Research Foundation, Brazil (grant no. 2016/00772-1.

## Conflicts of interest

The authors declare no conflicts of interest.

## References

[1] Noecker C., McNally C.P., Eng A., Borenstein E. (2017). High-resolution characterization of the human microbiome. Transl Res.

[2] Barko P.C., McMichael M., Swanson K.S., Williams D.A. (2018). The gastrointestinal microbiome: a review. J Vet Intern Med.

[3] Snelling A.M. (2005). Effects of probiotics on the gastrointestinal tract. Curr Opin Infect Dis.

[4] Stephenson M.F., Mfuna L., Dowd S.E., Wolcott R.D., Barbeau J., Poisson M. (2010). Molecular characterization of the polymicrobial flora in chronic rhinosinusitis. J Otolaryngol Head Neck Surg.

[5] Mahdavinia M., Keshavarzian A., Tobin M.C., Landay A.L., Schleimer R.P. (2016). A comprehensive review of the nasal microbiome in chronic rhinosinusitis (CRS). Clin Exp Allergy.

[6] Bokulich N.A., Chung J., Battaglia T., Henderson N., Jay M., Li H. (2016). Antibiotics, birth mode, and diet shape microbiome maturation during early life. Sci Transl Med.

[7] Depner M., Ege M.J., Cox M.J., Dwyer S., Walker A.W., Birzele L.T. (2017). A Bacterial microbiota of the upper respiratory tract and childhood asthma. J Allergy Clin Immunol.

[8] Aroniadis O.C., Brandt L.J. (2013). Fecal microbiota transplantation: past, present and future. Curr Opin Gastroenterol.

[9] Source: U.S. Food & Drug Administration, www.fda.gov/AboutFDA/Transparency/Basics/ucm361441.htm.

[10] Bojanova D.P., Bordenstein S.R. (2016). Fecal transplants: what is being transferred?. PLoS Biol.

[11] Oelschlaeger T.A. (2010). Mechanisms of probiotic actions — a review. Int J Med Microbiol.

[12] Cleland E.J., Drilling A., Bassiouni A., James C., Vreugde S., Wormald P.J. (2014). Probiotic manipulation of the chronic rhinosinusitis microbiome. Int Forum Allergy Rhinol.

[13] Cope E.K., Lynch S.V. (2015). Novel microbiome-based therapeutics for chronic rhinosinusitis. Curr Allergy Asthma Rep.

[14] Vancouver Sun. Believe it or snot: St. Paul's nasal mucus transplant study targets inflamed sinuses. Available at: https:https://couves-murcianas/news/local-news/believe-it--or-snot-st-pauls-nasal-mucous-transplant-study-targets-inflamed-sinuses [accessed 6 Feb 2019].

[15] Tapiovaara L., Pitkaranta A., Korpela R. (2016). Probiotics and the upper respiratory tract - a review. Pediatric Infect Dis.

[16] Abreu N.A., Nagalingam N.A., Song Y., Roediger F.C., Pletcher S.D., Goldberg A.N. (2012). Sinus microbiome diversity depletion and Corynebacterium tuberculostearicum enrichment mediates rhinosinusitis. Sci Transl Med.

[17] Mårtensson A., Greiff L., Lamei S.S., Lindstedt M., Olofsson T.C., Vasquez A. (2016). Effects of a honeybee lactic acid bacterial microbiome on human nasal symptoms, commensals, and biomarkers. Int Forum Allergy Rhinol.

[18] Mårtensson A., Abolhalaj M., Lindstedt M., Mårtensson A., Olofsson T.C., Vásquez A. (2017). Clinical efficacy of a topical lactic acid bacterial microbiome in chronic rhinosinusitis: a randomized controlled trial. Laryngoscope Investig Otolaryngol.

[19] Alromaih S., Mfuna Endam L., Desrosiers M., Cousineau B., Madrenas J. (2016). Self administered topical probiotic is beneficial for refractory CRS. Otolaryngol Head Neck Surg.

